# Moses Maimonides: Biographic Outlines

**DOI:** 10.5041/RMMJ.10002

**Published:** 2010-07-02

**Authors:** Fred Rosner

**Affiliations:** Teaching Attending Physician, Mount Sinai Services at Elmhurst Medical Center, Elmhurst, New York, USA; Professor of Medicine, Mount Sinai School Of Medicine, New York, USA

Moses, son of Maimon (*Rambam* in Hebrew, *Abu Imran Musa Ibn Maimun* in Arabic), was born in Cordova, Spain on 30 March 1135, corresponding to Passover eve of the Hebrew year 4895. His mother died in childbirth, and consequently his father Dayan (judge) Maimon raised him. Persecution by the Almochades, a fanatical group from North Africa, forced the Maimon family to flee Cordova in the year 1148. Maimonides was 13 years old. The family wandered through southern Spain and northern Africa for the next 10 years and finally settled in Fez, Morocco in 1158.

Little is known of Maimonides’ early life and medical education. There are no sources indicating that Maimonides had any formal medical education. In his *Medical Aphorisms* (see below), he mentions “the elders before whom I have read”; this is the only allusion to some semi-private study of medicine. A few times he mentions the son of Ibn Zuhr from whom he heard teaching of the latter’s illustrious father (the great physician Abu Marwan Ibn Zuhr), whom Maimonides held in great esteem.

Maimonides must have been an avid reader, since his medical writings show a profound knowledge of ancient Greek authors in Arabic translations and Moslem medical works. Hippocrates, Galen, and Aristotle were his Greek medical inspirations, and Rhazes of Persia, Al Farabi, and Ibn Zuhr, the Spanish-Arabic physician, are Moslem authors frequently quoted by Maimonides.

The Maimon family left Morocco in 1165, traveled to Palestine, landing in Acco, and from there to Egypt where they settled in Fostat (old Cairo). Maimonides turned to medicine as a livelihood only after the death of his father in 1166 and the death of his brother in a shipwreck shortly thereafter.

Maimonides was left with his brother’s wife and children to support and, after a year’s illness following his father’s death, entered into the practice of medicine. In 1174, at age 39, he was appointed Court Physician to Visier Alfadhal, Regent of Egypt, during the absence of the Sultan, Saladin the Great, who was fighting in the Crusades in Palestine. It was at this time that Richard the Lion-Hearted, also fighting in the Crusades, is reported to have invited Maimonides to become his personal physician, an offer which Maimonides declined. His reputation as a physician grew in Egypt and neighboring countries, and his fame as theologian and philosopher became worldwide.

In 1193 Saladin died, and his eldest son, Al Afdal Nur ad Din Ali, a playboy, succeeded him. As a result, Maimonides’ medical duties became even heavier as described in the famous letter he wrote to his friend, disciple, and translator, French Rabbi Samuel Ibn Tibbon, in the year 1199.

“… I live in Fostat and the Sultan resides in Cairo; these two places are two Sabbath limits [marked-off areas around a town within which it is permitted to move on the Sabbath; approximately one-and-a-half miles] distant from each other. My duties to the Sultan are very heavy. I am obliged to visit him every day, early in the morning, and when he or any of this children or concubines is indisposed, I cannot leave Cairo but must stay during most of the day in the palace. It also frequently happens that one or two of the officers fall sick and I must attend to their healing. Hence, as a rule, every day, early in the morning, I go to Cairo and, even if nothing unusual happens there, I do not return to Fostat until the afternoon. Then I am famished but I find the antechambers filled with people, Jews and Gentiles, nobles and common people, judges and policemen, friends and enemies,—a mixed multitude who await the time of my return.

“I dismount from my animal, wash my hands, go forth to my patients, and entreat them to bear with me while I partake of some light refreshment, the only meal I eat in twenty-four hours. Then I go to attend to my patients and write prescriptions and directions for their ailments. Patients go in an out until two hours and more into the night. I converse with them and prescribe for them even while lying down from sheer fatigue. When night falls, I am so exhausted that I can hardly speak.

“In consequence of this, no Israelite can converse with me or befriend me [on religious or community matters] except on the Sabbath. On that day, the whole congregation, or at least the majority comes to me after the morning service, when I instruct them as to their proceedings during the whole week. We study together a little until noon, when they depart. Some of them return and read with me after the afternoon services until evening prayer. In this manner, I spend the days. I have here related to you only a part of what you would see if you were to visit me…”

Maimonides was also the spiritual leader of the Jewish community of Egypt. At age 33, in the year 1168, shortly after settling in Fostat (old Cairo), he completed his first major work, the *Commentary on the Mishnah*. In 1178, 10 years later, his *magnum opus*, the *Mishneh Torah* was finished. This monumental work is a 14-book compilation of all Biblical and Talmudic law and remains a classic to this day. In 1190, Maimonides’ great philosophical masterpiece, the *Guide for the Perplexed*, was completed.

Maimonides died on 13 December 1204 (Tebeth 20, 4965 in the Hebrew calendar), and was buried in Tiberias. Legend relates that Maimonides’ body was placed upon a donkey and the animal set loose. The donkey wandered and wandered and finally stopped in Tiberias. That is the site where the great Maimonides (Rambam) was buried.

Maimonides was a prolific writer. We have already mentioned his famous trilogy, the *Commentary on the Mishnah*, the *Mishneh Torah*, and the *Guide for the Perplexed*. Each of these works alone would have indelibly recorded Maimonides’ name for posterity. However, in addition to these he also wrote a *Book on Logic* (*Ma’amar Hahigayon*), a *Book of Commandments* (*Sefer Hamitzvoth*), an *Epistle to Yemen* (*Iggereth Hashmad*), a *Treatise on Resurrection* (*Ma’amar Techiyath Hamethim*), commentaries on several tractates on the Talmud, and over 600 Responsa. Several additional works including the so-called Prayer of Maimonides^1^ are attributed to him but are, in fact, spurious, the prayer having been written in 1783.^1^,^2^

Over and above all the books we have just enumerated, Maimonides also wrote 10 medical works.^3^ The following is a brief examination and analysis of these medical writings. The first is called *Extracts from Galen*. Galen’s medical writings consist of over 100 books and required two volumes just to catalogue and index them all. Maimonides, therefore, extracted what he considered the most important of Galen’s pronouncements and compiled them verbatim in a small work which was intended primarily for the use of students of Greek medicine. This work, as all of Maimonides’ medical books, was originally written in Arabic. No complete Arabic manuscript exists today, but several Hebrew manuscript translations are available. This work has never been published in any language, but brief excerpts from there in both English and Hebrew appeared in a Hebrew periodical.^4^ A complete English translation is being prepared by Barzel.

The second of Maimonides’ writings is the *Commentary on the Aphorisms of Hippocrates*. The famous aphorisms of Hippocrates were translated from Greek into Arabic by Hunain Ibn Izchak in the ninth century. Maimonides wrote his commentary on this translation. Two incomplete Arabic manuscripts exist. A good medieval translation into Hebrew was made by Moses ben Samuel Ibn Tibbon. In this work, Maimonides occasionally criticizes both Hippocrates and Galen where either of these Greeks differs from his own view. For example, in chapter five, Hippocrates is quoted as having said, “ a boy is born from the right ovary, a girl from the left”, to which Maimonides remarks: “A man should be either prophet or genius to know this.” The introduction to this work was edited in the original Arabic with two Hebrew translations and one in German by Steinschneider in 1894.^5^ The entire work was published by Hasida in 1935^6^ and again in a definitive edition by Muntner in 1961.^7^ Bar Sela and Hoff have published Maimonides’ interpretation of the first aphorism of Hippocrates.^8^ This is the famous aphorism which has been called the motto or credo of the art of medicine: “Life is short, and the art long, the occasion fleeting, experience fallacious and judgment difficult. The physician must not only be prepared to do what is right himself, but must also make the patient, the attendant and the externals co-operate.”

The third of Maimonides’ medical works is the *Medical Aphorisms of Moses* (*Pirke Moshe*) and is the most voluminous of all. This book is comprised of 1,500 aphorisms based mainly on Greek medical writers. There are 25 chapters, each dealing with a different area of medicine including anatomy, physiology, pathology, symptomatology and diagnosis, etiology of disease and therapeutics, fevers, blood-letting or phlebotomy, laxatives and emetics, surgery, gynecology, hygiene, exercise, bathing, diet, drugs, and medical curiosities. A complete Arabic original manuscript exists in the Gotha library in the former East Germany. A Hebrew translation was made in the thirteenth century and published in Lemberg, Poland in 1834 and again in Vilna in 1888.^9^ The definitive Hebrew edition is that of Muntner dated 1959.^10^*Maimonides’ Aphorisms*^11^ were also translated into Latin in the thirteenth century and appeared as incunabulum in Bologna in 1489 and again in Venice in 1497, followed by several printed Latin editions.^12^ Only small fragments of this work have ever appeared in a Western language.^13^–^16^ A complete English version by Rosner and Muntner was published in two volumes^17^,^18^ and reprinted.^19^

A few excerpts from this most important work will give the reader the flavor of Maimonidean medical thinking. Maimonides speaks of cerebrovascular disease: “one can prognosticate regarding a stroke, called apoplexy. If the attack is severe, then he will certainly die but if it is minor, then a cure is possible, though difficult… the worst situation that can occur following a stroke is the complete irreversible suppression of respiration…”

Maimonides seems to be describing diabetes when the states: “Individuals in whom sweet white [humor] occurs are very somnolent [? Hyperglycemia]. To those who have an excess of sour white [humor] hunger occurs, then they become extremely thirsty. When this white liquid is neutralized, the thirst will disappear.” Maimonides explains that diabetes mellitus was seldom seen in “cold” Europe, whereas it was frequently encountered in “warm” Africa. He also reports this disease to be associated with the imbibition of suave water of the Nile. (Maimonides lived in Fostat or old Cairo.) There follows the English translation of this important aphorism no. 69 from the eighth chapter: “Moses says: I, too, have not seen it in the West [Spain, where Maimonides was born and/or Morocco where he fled from the persecution of the Almochades] nor did any one of my teachers under whom I studied mention that they had seen it [diabetes]. However, here in Egypt, in the course of approximately ten years, I have seen more than twenty people who suffer from this illness. This brings one to the conclusion that this illness occurs mostly in warm countries. Perhaps the waters of the Nile, because of their suaveness, may play a role in this.”

A very accurate description of obstructive emphysema is provided during a lengthy discussion of respiratory disease: the “…reason [for respiratory embarrassment] is narrowing of the organs of respiration, and then the breast is seen to greatly expand. This expansion produces rapid and cut-off [respirations]…”

Clubbing of the fingers associated with pulmonary disease, already described by Hippocrates, is beautifully depicted: “With an illness affecting the lungs called ‘hasal’, namely phthisis, there develops rounding of the nail as a rainbow.” The signs and symptoms of pneumonia are remarkably accurately described: “The basic symptoms which occur in pneumonia and which are never lacking are as follows: acute fever, sticking [pleuritic] pain in the side, short rapid breaths, serrated pulse and cough, mostly [associated] with sputum…” Hepatitis is just as beautifully described: “the signs of liver inflammation are eight in number as follows: high fever, thirst, complete anorexia, a tongue which is initially red and then turns black, biliary vomitus, initially yellow egg-yolk in color which later turns dark green, pain of the right side which ascends up to the clavicle… Occasionally a mild cough may occur and a sensation of heaviness which is first felt on the right side and then spreads widely…”

So much for the medical aphorisms of Moses Maimonides.

The fourth of Maimonides’ medical writings is his *Treatise on Hemorrhoids*. This work was written for a nobleman, as Maimonides describes in the introduction, probably a member of the Sultan’s family. There are seven chapters dealing with normal digestion, foods harmful to patients with hemorrhoids, beneficial foods, general and local therapeutic measures such as sitz baths, oils, and fumigations. Maimonides disapproves of blood-letting or surgery for hemorrhoids except in very severe cases. Maimonides’ whole approach to the problem seems to bespeak a modern medical trend. The *Treatise on Hemorrhoids* was first published by Kroner in 1911 in Arabic, Hebrew, and German.^20^ A general description of the work in English appeared in 1927 by Bragman.^21^ The definitive Hebrew edition is that of Muntner dated 1965,^22^ and an English translation of the entire work was published by Rosner and Muntner.^23^

In the introduction to this work, Maimonides describes the reason for writing it:

“There was a youth, [descended] from knowledgeable, intelligent and comprehending forebears, from a prominent and renowned family, distinguished and charitable and of great means, in whom the affliction of hemorrhoids occurred at the mouth of the rectum, that interested me in his problem and placed the task [of healing them] upon me. These irritated him on some occasions and he treated them in the customary therapeutic manner until the pain subsided and the prolapsed rectum [literally: excesses that protruded] became reduced and retuned to the interior of the body so that his [bodily] functions returned to normal. Because this [illness] recurred many times, he considered having them extirpated in order to uproot this malady from its source so that it not return again. I informed him of the danger inherent in this, in that it is not clear if these hemorrhoids [literally: additions] are of the variety which should be excised or not, since there are people in whom they have once been [surgically] extirpated and in whom other hemorrhoids develop. This is because the causes which gave rise to the original ones remained and, therefore, new ones develop.”

Here Maimonides provides an insight into the etiology of disease in general in that he regards operative excision of hemorrhoids with skepticism, because surgery does not remove the underlying causes which produced the hemorrhoids in the first place.

The fifth work is Maimonides’ *Treatise on Cohabitation* written for the nephew of Saladin, the Sultan al Muzaffar Umar Ibn Nur Ad-Din. The Sultan indulged heavily in sexual activities and asked Maimonides, his physician, to aid him in increasing his sexual potential. The work consists mainly of recipes of foods and drugs which are either aphrodisiac or anti-aphrodisiac in their actions. Maimonides advises moderation in sexual intercourse and describes the physiology of sexual temperaments. There are two versions of this book, a short authentic and a longer spurious version. Both were first edited and published by Kroner in 1906 in Hebrew and German.^24^ Ten years later, Kroner published the true short version from the original Arabic manuscripts in Granada.^25^ An Italian edition appeared in 1906,^26^ and English^27^ and Spanish^28^ translations were published in 1961. The definitive Hebrew edition of both authentic^29^ and spurious^30^ versions of Maimonides’ book on sex is that of Muntner dated 1965. A new English translation of the true work by Rosner has been published.^31^

The sixth medical book of Moses Maimonides is his *Discourse on Asthma*. The patient for whom this book was written suffered from violent headaches which prevented him from wearing a turban. The patient’s symptoms began with a common cold, especially in the rainy season, forcing him to gasp for air until phlegm was expelled. The patient asked whether a change of climate might be beneficial. Maimonides, in 13 chapters, explains the rules of diet and climate in general and those rules specifically suited for asthmatics. He outlines the recipes of foods and drugs and describes the various climates of the Middle East. He states that the dry Egyptian climate is efficacious for sufferers from this disease and warns against the use of very powerful remedies. Several Arabic, Hebrew, and Latin manuscripts exist.^32^ The first critical edition of this work appeared in Hebrew in 1940, edited by Muntner.^33^ Additional manuscripts became available after World War II, and a corrected, improved, and revised, second Hebrew edition appeared in 1963.^34^ Only 300 copies of this edition were printed, and thus a third edition was published by Muntner in 1965.^35^ An English version of Maimonides’ book on asthma was published in 1963^36^ and a French translation in 1965.^37^

The last chapter of this work deals with concise admonitions and aphorisms which Maimonides considered “useful to any man desirous of preserving his health and administering to the sick.” The chapter begins as follows: “The first thing to consider … is the provision of fresh air, clean water and a healthy diet.” Fresh air is described in some detail: “… City air is stagnant, turbid and thick, the natural result of its big buildings, narrow streets, the refuse of its inhabitants … one should at least choose for a residence a wide-open site … living quarters are best located on an upper floor … and ample sunshine … Toilets should be located as far as possible from living rooms. The air should be kept dry at all times by sweet scents, fumigation and drying agents. The concern for clean air is the foremost rule in preserving the health of one’s body and soul.” Let our air pollution control programmers take cognizance of Maimonides’ prophetic statements nearly 800 years ago.

The seventh medical work of Maimonides is his *Treatise on Poisons and Their Antidotes*, which is the subject of the present book. It is one of the most interesting and popular works because it is very scientific and modern in its approach and was, therefore, used as a text-book of toxicology throughout the Middle Ages.

The numerous extant Arabic, Hebrew, and Latin manuscripts^38^ are described in the bibliography chapter below. A German translation was published in 1873 by Steinschneider.^39^ A French translation appeared in 1865 by Rabbinowicz and was reprinted in 1935.^40^ An English translation of Steinschneider’s German version is that of Bragman in 1926.^41^ The definitive Hebrew edition of Muntner appeared in 1942^42^, and Muntner’s English version was published in 1966;^43^ these translations, their strengths and their deficiencies, are discussed in detail elsewhere.^44^

The eighth book is the *Regimen of Health* (*Regimene Sanitatis*). Maimonides wrote it in 1198 during the first year of the reign of Sultan Al Malik Al Afdal, eldest son of Saladin the Great. The Sultan was a frivolous and pleasure-seeking man of 30, subject to fits of melancholy or depression due to his excessive indulgences in wine and women, and his warlike adventures against his own relatives and in the Crusades. He complained to his physician of constipation, dejection, bad thoughts, and indigestion. Maimonides answered his royal patient in four chapters. The first chapter is a brief abstract on diet taken mostly from Hippocrates and Galen. The second chapter deals with advice on hygiene, diet, and drugs in the absence of a physician. The third extremely important chapter contains Maimonides’ concept of “a healthy mind in a healthy body”, perhaps the first description of psychosomatic medicine. He indicates that the physical well-being of a person is dependent on his mental well-being and vice versa. The final chapter summarizes his prescriptions relating to climate, domicile, occupation, bathing, sex, wine-drinking, diet, and respiratory infections.

The whole treatise on the *Regimen of Health* is short and concise but to the point. This is the reason for its great success and popularity throughout the years. It is extant in numerous manuscripts. A Hebrew translation from the original Arabic was made by Moses ben Samuel Ibn Tibbon in 1244, and this version was reprinted several times in the nineteenth century (Prague 1838, Jerusalem 1885, Warsaw 1886). Two Latin translations were made in the thirteenth century. Several fifteenth-century incunabulae and sixteenth-century editions of these Latin versions exist. A French translation by Carcousse appeared in 1887 in Algiers.^44^ The Arabic text with German and Hebrew translations was published by Kroner in 1925,^45^ although he had already published the all-important chapter three dealing with psychosomatic medicine 11 years earlier in 1914.^46^ An English translation of chapter three by Bragman appeared in 1932.^47^ The definitive Hebrew edition is that of Muntner dated 1957.^48^ Two English translations of the entire work were published: in 1958 by Gordon^49^ and in 1964 by Bar Sela, Hoff, and Faris.^50^ Another German translation by Muntner appeared in 1966.^51^ These numerous editions in many languages attest to the importance and popularity of Maimonides’ *Regimen of Health*.

The ninth medical writing of Maimonides is the *Discourse on the Explanation of Fits*. This work has been called Maimonides’ swan-song as it probably was the last of his medical works, having been written in the year 1200, four years before his death. It was also written for the Sultan Al Malik Al Afdal and is sometimes considered to represent chapter five of the *Regimen of Health*. The Sultan persisted in his over-indulgences and wrote to Maimonides, who was himself ill, asking advice about his health. Maimonides confirms most of the prescriptions of the Sultan’s other physicians regarding wine, laxatives, bathing, exercise, and the like, and near the end gives a very detailed hour-by-hour regimen for the daily life of the Sultan. The original Arabic was edited and published with Hebrew and German translations by Kroner in 1928.^52^ English editions by Bar Sela, Hoff, and Faris in 1964^50^ and Rosner and Muntner in 1969,^23^ another German version by Muntner in 1966,^51^ and another Hebrew edition by Muntner in 1969^53^ are available. The most recent edition is that by Leibowitz and Marcus entitled *On the Causes of Symptoms*,^54^ in which the text is presented in four languages (Arabic, Hebrew, Latin, and English) and is accompanied by a running commentary, explanatory essays, and a comprehensive catalogue of drugs.

The final authentic medical book of Maimonides is the *Glossary of Drug Names*. This work was discovered by Max Meyerhof, an ophthalmologist in Egypt, in the Aya Sofia library in Istanbul, Turkey as Arabic manuscript no. 3711. Dr Meyerhof edited the original Arabic and provided a French translation with a detailed commentary, which he published in 1940 in Cairo.^55^ A Hebrew edition by Muntner appeared in 1969,^53^ and an English translation by Rosner was published in 1979.^56^ The work is essentially a pharmacopoeia and consists of 405 short paragraphs containing names of drugs in Arabic, Greek, Syrian, Persian, Berber, and Spanish.

In summary, Maimonides’ medical writings are varied, comprising extracts from Greek medicine, a series of monographs on health in general and several diseases in particular, and a recently discovered pharmacopoeia demonstrating Maimonides’ extensive knowledge of Arabic medical literature and his familiarity with several languages. Some people feel that Maimonides’ medical writings are not as original as his theological and philosophical writings. However, his medical works demonstrate the same lucidity, conciseness and formidable powers of systematization and organization so characteristic of all his writings. The *Treatise on Poisons and Their Antidotes* the *Regimen of Health*, and the *Medical Aphorisms of Maimonides* became classics in their fields in medieval times.

I would like to conclude by citing a paragraph from my first paper on Maimonides.^57^

“Maimonides died on 13 December 1204 (Tebeth 20, 4965, in the Jewish calendar) and was buried in Tiberias, Palestine… The Christian, Moslem and Jewish worlds mourned him. His literary ability was incredible and his knowledge encyclopedic. He mastered nearly everything known in the fields of theology, mathematics, law, philosophy, astronomy, ethics, and, of course, medicine. As a physician, he treated disease by the scientific method, not by guesswork, superstition, or rule of thumb. His attitude towards the practice of medicine came from his deep religious background, which made the preservation of health and life a divine commandment. His inspiration lives on through the years and his position as one of the medical giants of history is indelibly recorded. He was physician to Sultans and Princes, and as Sir William Osler said, ‘he was Prince of Physicians’. The heritage of his great medical writings is being more and more appreciated. To the Jewish people he symbolized the highest spiritual and intellectual achievement of man on this earth; as so aptly stated, ‘from Moses to Moses there never arose a man like Moses’, and none has since.”
